# Development and evaluation of a high-throughput, low-cost genotyping platform based on oligonucleotide microarrays in rice

**DOI:** 10.1186/1746-4811-4-13

**Published:** 2008-05-29

**Authors:** Jeremy D Edwards, Jaroslav Janda, Megan T Sweeney, Ambika B Gaikwad, Bin Liu, Hei Leung, David W Galbraith

**Affiliations:** 1 University of Florida, Gulf Coast Research & Education Center, Wimauma FL, 33598, USA; 2 University of Arizona, Department of Plant Sciences and Bio5 Institute for Collaborative Bioresearch, Tucson AZ, 85721, USA; 3 National Research Centre on DNA Fingerprinting, National Bureau of Plant Genetic Resources, New Delhi, India; 4 Guangdong Academy of Agricultural Sciences (GDAAS), Guangdong, Pr China; 5 International Rice Research Institute (IRRI), Los Banos, The Philippines

## Abstract

**Background:**

We report the development of a microarray platform for rapid and cost-effective genetic mapping, and its evaluation using rice as a model. In contrast to methods employing whole-genome tiling microarrays for genotyping, our method is based on low-cost spotted microarray production, focusing only on known polymorphic features.

**Results:**

We have produced a genotyping microarray for rice, comprising 880 single feature polymorphism (SFP) elements derived from insertions/deletions identified by aligning genomic sequences of the *japonica *cultivar Nipponbare and the *indica *cultivar 93-11. The SFPs were experimentally verified by hybridization with labeled genomic DNA prepared from the two cultivars. Using the genotyping microarrays, we found high levels of polymorphism across diverse rice accessions, and were able to classify all five subpopulations of rice with high bootstrap support. The microarrays were used for mapping of a gene conferring resistance to *Magnaporthe grisea*, the causative organism of rice blast disease, by quantitative genotyping of samples from a recombinant inbred line population pooled by phenotype.

**Conclusion:**

We anticipate this microarray-based genotyping platform, based on its low cost-per-sample, to be particularly useful in applications requiring whole-genome molecular marker coverage across large numbers of individuals.

## Background

Considerable interest exists in the ability to determine genotypes within species in a cost-effective manner. Cost-effectiveness is principally determined by desired outcome: when the outcome is a complete genotypic description of a single individual (for example a human patient), the cost is largely defined by healthcare economics, and is the driving force behind initiatives to minimize the whole genome costs of sequencing [1]. For outcomes in the agricultural sector, for example ones leading to identification of genes responsible for desired agronomic traits, genotyping is applied to large populations rather than single individuals, which considerably changes the economic considerations. Moreover, since downstream gene mapping and identification technologies are increasingly well-established for different crop species [2], the required resolution of such genotyping platforms need not approach the single-nucleotide level provided by whole genome sequencing. Consequently, economic considerations and practical applications of a genotyping technology are driven largely by cost-per-individual rather than cost-per-datum.

Microarray-based technologies for genotyping have become increasingly popular since they offer an assay that is highly multiplexed, and this was immediately recognized as providing a low cost per data point [3]. One of the earliest reports of microarray-based genotyping employed high density whole-genome tiling arrays, produced by photolithographic synthesis (Affymetrix, Santa Clara, CA), for the simultaneous discovery and assay of DNA polymorphisms in yeast. In genotyping assays based on microarrays, allelic variations are detected as differential hybridization of labeled genomic DNA to individual probes, or sets of probes, covering identifiable genomic locations. Using this approach, a large number of single feature polymorphisms (SFPs) were identified between two laboratory strains of yeast [4]. In this case, 3,714 markers were identified using microarrays which comprised 157,112 overlapping 25-mers spanning all annotated *Saccharomyces cerevisiae *open reading frames [5]. For the larger and more complex Arabidopsis genome, tiling arrays were not available, and hence the first experiments involved hybridization of labeled genomic DNA using Affymetrix AtGenome1 GeneChips based on available, expression-based annotation for open reading frames (ORFs). Despite this ORF-based focus, nearly 4,000 SFPs were identified between the Columbia (Col) and *Landsberg erecta *(L*er*) accessions [6]. In a subsequent study, more than 8000 SFPs were identified using the ATH1 GeneChip comprising 22,500 probe sets representing approximately 24,000 genes [7].

High density microarray platforms of this type provide a very large amount of information from single individuals, and therefore are ideally suited for polymorphism discovery [8] or for genome-wide association studies [9,10]. However, for genotyping populations, the economic utility of microarray genotyping platforms is a function not simply of the multiplexing level, but also of the costs associated with processing each sample [11]. Affymetrix Genechips have the conspicuous disadvantage of a high cost of production and hybridization per array, and this limits their use in situations requiring the genotyping of large numbers of individuals, such as in plant breeding. In contrast, the production of microarray slides through robotic printing of array elements is relatively inexpensive [12,13]. For microarrays of this type, the array elements (probes) are either PCR amplicons [14], or synthesized single-stranded oligonucleotides [15]. Since very little DNA is needed for printing each element, beyond the initial cost of production, the cost per element becomes vanishingly small. A further cost-savings is achieved since the microarrays are conventionally hybridized to mixed pairs of nucleic acid targets, separately labeled with different fluorochromes, rather than using one target per hybridization as done with Affymetrix Genechips.

Diversity array technology (DArT) is a modification of the amplified fragment length polymorphism (AFLP) procedure using a microarray platform [16-18]. In DArT, a pool of DNA fragments is produced from a subset of the genome by restriction enzyme digestion of genomic DNA followed by ligation of adaptors and PCR amplification with adaptor specific primers. Fragments from this pool of DNA are cloned and spotted on a microarray. Pools of target DNA are similarly generated from other samples, fluorescently labeled, and hybridized to the arrays. The assay reveals whether the specific cloned DNA fragments are present in the queried sample. An advantage of the DArT technology is that prior genome sequence information is not required; therefore it can be applied to a large range of species. A disadvantage is that, similarly to AFLP, the differential PCR amplification of specific fragments may vary between experiments depending on PCR conditions. Another disadvantage is that the sequence and precise genomic location of the cloned fragments is not known. Therefore, with DArT, it is difficult to target specific genes or genomic regions with higher densities of markers.

Here we describe and validate a method for cost-effective genotyping using printed microarrays comprising single-stranded oligonucleotide array elements. The microarrays were designed to recognize known polymorphic sequences. Each oligonucleotide probe corresponds to an insertion/deletion (indel) polymorphism (i.e. a SFP) discovered through the alignment of whole genome sequences. The DNA sequences used as probes were selected for uniqueness, and to have a uniform melting temperature, and a similar length (approximately 70 nucleotides), to ensure specificity of hybridization. Rice (*Oryza sativa*) was selected, because of the availability of whole genome sequences for the highly divergent *japonica *(International Rice Genome Sequencing Project ) and *indica *[19] cultivars. We recognized that it should be relatively straightforward to employ genomic sequence alignment to identify polymorphisms. Further, rice has abundant mapping populations and germplasm collections to which the genotyping technology can be applied. Finally, rice is considered world-wide the most important agricultural crop, because it provides approximately 23% of the caloric requirements of humans and up to 60% of the calories in countries that rely on rice as the main staple [20]. Because most of the rice improvement efforts occur in developing countries; a low-cost and robust method would be particularly important for breeding institutions with modest levels of research infrastructure.

This low-cost, focused method of genotyping, using printed long-oligonucleotide microarrays, will be particularly useful for applications that require high-density molecular marker coverage of entire genomes for large numbers of samples. Such applications include quantitative trait locus (QTL) mapping, genetic diversity and population structure studies, association mapping, molecular breeding, polymorphism surveys, and marker assisted selection. In this study, we describe the development and use and validation of the genotyping microarrays, and their utilization in the assessment of the levels of polymorphism and genetic relationships within a collection of diverse rice accessions, and to map a major gene conferring resistance to the rice blast pathogen (*Magnaporthe grisea*) in a segregating recombinant inbred line (RIL) population. Finally, since this method of genotyping is general in scope and can be implemented in other species, provided that sufficient genomic sequences from multiple individuals are available for the identification of SFPs, we describe a bioinformatics pipeline that has been developed for this purpose.

## Results

### Polymorphism discovery

**Figure 1:**
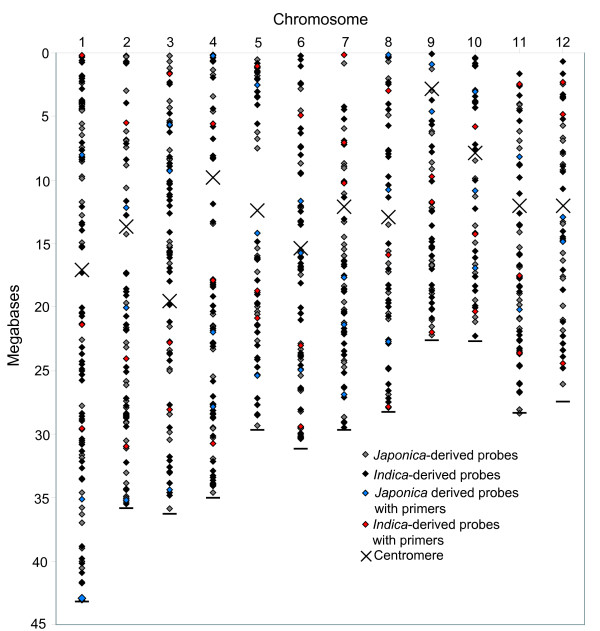
**Positions of SFP markers on the rice pseudomolecule map**. SFPs with oligonucleotide sequences complementary to Nipponbare are shown as black and those complementary to 93-11 are shown in grey. SFPs confirmed by PCR are shown in blue (Nipponbare) and red (93-11). The positions of the centromeres are indicated with an "X".

To create probes that can detect the presence or absence of indel sequences in a genomic DNA sample, the probes need to be complementary to a unique single copy sequence. To identify suitable indels, the genome sequences of the *japonica *rice cultivar Nipponbare and the *indica *cultivar 93-11 were aligned. Whole-genome alignments were done using MUMmer and NUCmer 3.18 [21-23]. The indel sequences were masked for simple repeats and for known rice repetitive elements [24]. Indels with at least 29 nucleotides of unique sequence were considered for probe design. The alignment of cv. Nipponbare and cv. 93-11 genomic sequences revealed 880 indel loci where oligonucleotide probes could be designed with specifications suitable for hybridization (Additional File 1). The indels used for probe design ranged from 29 to 426 nucleotides in length, with an average indel size of 76 nucleotides. Of the 880 probes designed, 423 are complementary to the Nipponbare allele and 457 are complementary to the 93-11 allele. The order and positions of the resulting SFP markers was determined based their positions in the cv. Nipponbare pseudomolecule assembly (Figure 1). The median distance between SFP markers is 234 kilobases, and the largest gaps occur around the locations of the centromeres [25] and in pericentromeric regions.

### Experimental verification of SFPs

**Figure 2:**
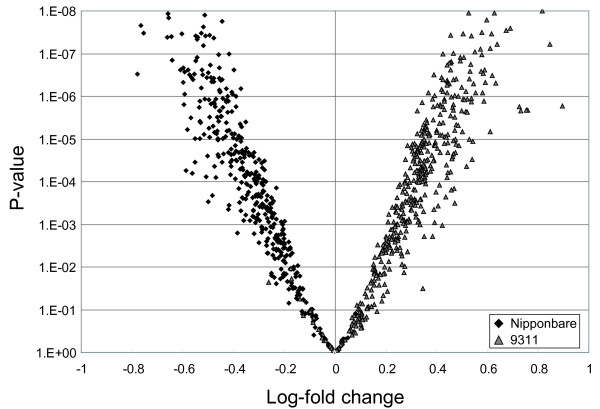
**A volcano plot comparing Nipponbare and 93-11 genomic DNA hybridizations with the 880 SFPs**. SFPs with oligonucleotide sequences complementary to Nipponbare are shown as black and those complementary to 93-11 are shown as grey.

To validate the genotyping protocol and the computationally predicted SFPs, we hybridized Cy5 and Cy3 labeled genomic DNA of cv. Nipponbare and cv. 93-11 to four microarrays, with each of the probes printed in triplicate. To be useful as molecular markers, the SPFs should be reliable and have a fold change between samples that is readily detectable. At a p-value of 0.05 or less, 676 probes (76.8%) had significant color-ratio fold changes in the predicted direction, and only four probes with fold changes opposite to that predicted (Table 2, Figure 2). We designed primers around the probes which showed fold changes in the opposite direction and were able to show that the predicted indel was present in each case. Of the 880 probes, 115 were unusable because they were considered to be "not found" based on more than 50% of the spots having a signal to noise ratio < 1 across all replicates. A further 19 probes were unusable because the signal intensities were saturated in more than 50% of the spots across replicates. The probes considered as being both found and unsaturated had a mean GC content of 46.8%. The probes considered saturated had a significantly higher (p-value = 1.86E-07) GC content of 53.6% and the probes considered not found had a significantly lower (p-value = 6.57E-09) GC content of 43.6%. The SPFs were also validated on slides configured in the 24-plex format using the restriction-ligation labeling procedure. Using this method, across eight arrays, 30 probes were considered to be not found, and 35 were considered to be saturated.

**Table 2:** Validation of SFPs for significant fold change (< 0.05), fold change in the predicted direction, and detection or saturation of hybridization signal.

**Category**	**Complementary to 93-11**	**Complementary to Nipponbare**	**Total**
Correct-significant	345	331	676
Correct-not significant	34	18	52
Incorrect-significant	4	0	4
Incorrect-not significant	10	4	14
Saturated	10	9	19
Not found	54	61	115

**Total**	**457**	**423**	**880**

To determine if the SFPs identified in this study could be used as individual PCR based markers, we attempted to design primers surrounding a selected subset (72) of the indels, 36 having deletions in Nipponbare and 36 having deletions in 93-11 (Figure 1, Additional File 1). Two of SFPs were located in highly repetitive regions and no unique primer sequences could be created to amplify only these loci. One SFP was surrounded by sequence that was highly diverged between Nipponbare and 93-11 such that no primers complementary to sequences conserved between Nipponbare and 93-11 could be created. We successfully designed primers surrounding the remaining 69 SFPs and, using these primers on Nipponbare and 93-11 genomic DNA, demonstrated that these loci could be individually assayed using PCR. This enables researchers to assay population polymorphisms more efficiently, either using a single hybridization to the genotyping microarray to define the polymorphic markers, and then employing only these markers for PCR-based analyses of populations, or screening large fine-mapping populations with only those markers flanking a QTL previously identified by microarray genotyping. Several of the amplicons showed a larger difference in allele size than predicted. We determined that this was due to the presence of highly repetitive sequence within the indel which was masked during the SFP discovery process.

### Polymorphisms within *O. sativa*

**Figure 3:**
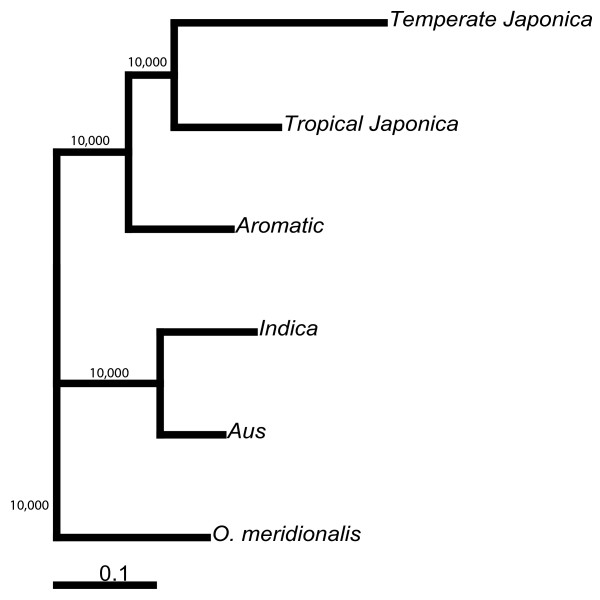
**Neighbor Joining tree using the SFP marker data to show the genetic relationships between the five sub-populations of rice**. Neighbor joining trees were constructed using four accessions per subpopulation. The bootstrap values are out of 10,000.

**Figure 4:**
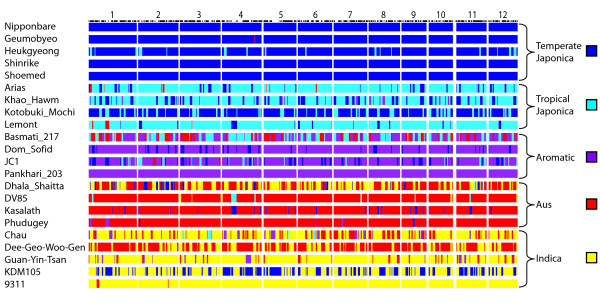
**Site-by-site probabilities for the population of origin of alleles across the twelve chromosomes of each accession**. There are four accessions per sub-population plus Nipponbare and 93-11 reference sequences. Calculations are done using the linkage model of STRUCTURE.

To be generally useful genetic markers, the SFPs between Nipponbare and 93-11 should be polymorphic between other *O. sativa *varieties. The genotyping microarrays were used to assess the polymorphisms across 20 diverse *O. sativa *varieties representing five sub-populations as determined through STRUCTURE [26] analysis with 169 microsatellite markers [27]. The genotype scores are listed in Additional File 2. Average levels of polymorphism for pairs of varieties were calculated within and between sub-populations (Table 1). The highest levels of polymorphism (66.2%) were found between the *temperate japonica *and *indica *sub-populations. This is expected, given that the sequenced varieties used for SFP discovery, Nipponbare and 93-11, belong to the *temperate japonica *and *indica *sub-populations respectively. The lowest level of polymorphism was within the *temperate japonica *sub-population (10.4%) which is also the least diverse sub-population according to microsatellite markers [27]. Using the SFP genotype data, a neighbor-joining tree was constructed to examine the genetic relationships between the five subpopulations (Figure 3). The relationships according to the SFP analysis are concordant with previous studies [27-30] with extremely high bootstrap support. Model-based clustering using STRUCTURE was used to calculate site-by-site probabilities of sub-population origin of alleles across the twelve chromosomes for each of the 20 *O. sativa *varieties (Figure 4). The clustering using the SFP data is consistent with the clustering using microsatellite markers. Further, the use of high density SFP markers resolves large blocks of chromosomes with ancestry that differs from the overall sub-population assignment of the individuals.

**Table 1:** Average pairwise percent of polymorphic markers between accessions belonging to the five rice subpopulations. The percent of polymorphic markers is calculated using four accessions per sub-population.

**Cross**	**Mean Polymorphism**
*Temperate Japonica *X *Temperate Japonica*	10.4%
*Temperate Japonica *X *Tropical Japonica*	20.1%
*Temperate Japonica *X *Aromatic*	24.7%
*Temperate Japonica *X *Aus*	37.1%
*Temperate Japonica *X *Indica*	46.8%
*Tropical Japonica *X *Tropical Japonica*	16.3%
*Tropical Japonica *X *Aromatic*	23.3%
*Tropical Japonica *X *Aus*	31.8%
*Tropical Japonica *X *Indica*	40.2%
*Aromatic *X *Aromatic*	18.0%
*Aromatic *X *Aus*	29.8%
*Aromatic *X *Indica*	38.8%
*Aus *X *Aus*	18.0%
*Aus *X *Indica*	27.1%
*Indica *X *Indica*	25.6%

### Bulked Segregant Analysis with SFPs

**Figure 5:**
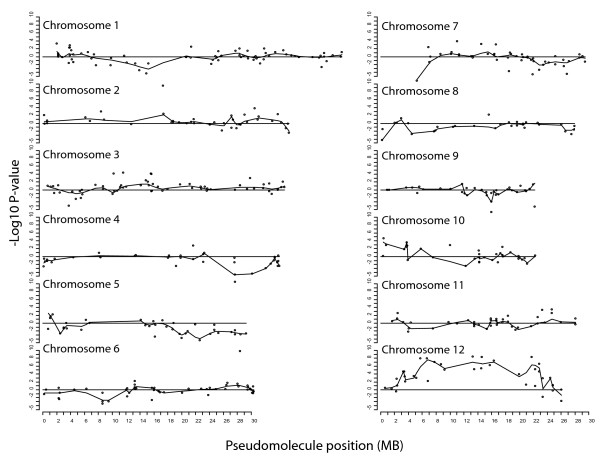
**Significance of SFP fold change measurements between pools of DNA from blast resistant and susceptible RILs**. SFP markers showing association of the resistant parent (SHZ) allele with the resistant pool are assigned positive values and association with the susceptible parent allele (LTH) with the resistant pool are assigned negative values. The line is drawn using loess smoothing.

The SFP genotyping microarrays were used in a bulk segregant analysis [31] experiment with a RIL population segregating for resistance to a single isolate (IsoIV) of rice blast disease. Pools of 73 resistant and 73 susceptible lines were hybridized to six slides using a balanced dye-swap design. SFPs that are linked to the gene(s) conferring resistance should display significant differences in color ratios reflecting differences in allele frequencies between the two pools. SFPs that are unlinked should have balanced color ratios. The ANOVA method was used to calculate p-values for each SFP marker. The log transformed fold changes of the SFPs and the SFPs positions were then plotted on the pseudomolecule assembly (Figure 5). In this figure, we employed the convention that the p-values were plotted in a positive direction if the direction of the ratio signified greater representation of the SHZ (resistant parent) in the resistant pool, and in the negative direction for SFPs signifying greater representation of the LTH (susceptible parent) alleles in the resistant pool. A cluster of SFPs with significant p-values was found on chromosome 12, indicating that SHZ alleles in this chromosomal region may confer resistance. The top 10 most significant SFPs all fall within the same region of chromosome 12, and are ordered by most to least significant (Table 4) with Benjamini and Hochberg [32] adjusted p-values. The gene conferring resistance to the blast IsoIV isolate designated as Pi-GD-3(t) has previously been mapped using microsatellite markers in the same RIL lines [33]. The resistance gene was most closely linked to microsatellite marker RM179 on chromosome 12 close to the most significant SFP (adjusted p-value 5.06E-09) at position 13266396. Thus, the SFP bulked segregant results are consistent with previous genetic mapping based on conventional microsatellite markers.

## Discussion

Alignment of genomic sequence was demonstrated to be an accurate method for *in silico *prediction of SFPs. Additional SFP markers could be obtained using the same pipeline for indel discovery and probe design with the input of genomic sequences of other rice varieties or related species as they become available. SFPs may also be discovered in other rice varieties through hybridization of genomic DNA to tiling arrays [6]. The sequences of the probes identified as polymorphic on the tiling arrays could be used to design 70-mer probes to be included on the lower cost spotted microarrays. Using this approach, efforts are currently underway to expand the number and varietal sources of SFP markers on the genotyping microarray. The on-going whole-genome SNP discovery project in rice is expected to generate information on distribution of SNP across 20 lines using Nipponbare sequence as the reference [34]. Although the Perlegen hybridization approach will primarily yield SNP data, results from Arabidopsis suggest that small to medium size indels could also be inferred from the hybridization data file, providing a rich source of deletion sites across diverse germplasm for designing SNP markers [35] and (D. Weigel, personal communication). The described methods for SFP discovery and the microarray-based genotyping assay could also be implemented in any other species having genomes small enough (e.g., medicago, sorghum, soybean, and tomato) to permit adequate levels of hybridization with high specificity to the spotted probes. However, cross-hybridization problems may prevent the use of a microarray-based genotyping methods in polyploids or species with large genomes. The microarray-based genotyping platform is particularly useful for genetic mapping applications requiring whole-genome scans.

Of the various possible applications to gene mapping, we successfully demonstrated bulked-segregant analysis where the use of genotyping microarrays is advantageous because it provides a quantitative assessment of allele frequencies between groups of pooled samples. By pooling genotypes of two phenotypic extremes, these experiments can be accomplished rapidly using a small number of microarrays. We showed that we could pool large number of genotypes per extreme (73 in our case), thereby defining a narrow genetic window. The median spacing of SFP markers on the chromosomes is 234 kb. Assuming a 50% polymorphism, BSA mapping provides resolution of about 0.5 Mb or approximately 2 cM. Once the location is mapped, simple sequence repeat (SSR) markers can be used to saturate the region. Applying the same approach, we were able to rapidly define the chromosomal location of a mutation (M. Bernardo and H. Leung, unpublished data).

For conventional QTL mapping, the low cost per individual of this assay enables genotyping of large segregating populations. Our current protocols, including replication, have reduced the cost per genotype to less than $18 (Low cost labeling method presented in Additional File 4). The rice genotyping microarrays will be available for distribution through the Galbraith lab  High density molecular marker coverage of QTL mapping populations will delimit recombination breakpoints with greater precision and potentially enhance the mapping resolution. Molecular breeding applications are also likely to benefit from microarray-based genotyping. In backcrossing experiments, the whole-genome coverage would allow genotypic positive selection for the desired alleles at specific loci and negative selection against "background" donor alleles at all other loci throughout the genome [36]. The whole-genome coverage also provides the ability to construct "graphical genotypes" to more efficiently pyramid desired alleles at multiple loci [37]. Microarrays may also be used to genotype introgression lines derived from wide crosses. Tracking of introgressed chromosomal segments with high precision in introgression lines combined with phenotypic analysis can be used to establish phenotypic effects associated with particular introgressions through advanced backcross QTL analysis [38] and provide opportunities for cloning of the underlying genes. Collections of introgression lines genotyped at a high resolution will facilitate more efficient utilization of genetic resources [39,40]. While SNP platforms for rice are being developed which will allow the generation of large amounts of data for pennies a data point, they are still more than a hundred dollars per sample, which limits their use in mapping populations and other applications in which a large number of samples need to be run.

A method similar to the one described here has been implemented in Arabidopsis (Salathia et al, 2007). Our methods offer the advantage of a labeling procedure with a substantial reduction in the per-sample cost, and we provide a flexible platform for SFP identification and probe design that can be used with genome sequences in any other species.

The ability to detect polymorphisms across diverse rice varieties makes the microarray-based genotyping platform useful for population genetics studies. Domesticated crops have complicated genetic histories and individual can have a complex network of genetic relationships. The microarray platform can produce a density of genotypic data that is sufficient to track sub-population ancestry across chromosome segments using structure analysis [26]. The resulting population structure information could be used to a framework for association mapping with diverse lines [41-43] or elite lines [19,44]. Microarray-based genotyping could also be used in plant variety protection as a method to identify and distinguish between released varieties with the robustness afforded by large numbers of molecular markers.

## Conclusion

Variations in DNA sequence are a mixture of SNPs and indels. Indels are generated by different mechanisms than SNPs. Indels may arise through transposon mediated rearrangements [45,46] and genomic expansion and contraction [47]. Indels within or spanning genes or regulatory regions can be a significant component of intraspecific genetic variation and a potential source of hererosis [48]. The flexibility of the spotted arrays also makes it relatively simple to add more SFP markers over time to increase the coverage of individual chromosomes. The results gathered so far suggest that this SFP-based platform should provide a very useful complement to SNP terminologies for associating DNA sequence polymorphisms with phenotypic variation.

## Methods

### Polymorphism discovery

The genotyping platform employs oligonucleotide probes to detect the presence or absence of indel sequences in a genomic DNA sample. Each indel must therefore contain a stretch of unique, single copy sequence so that this is the only sequence in the genome that can hybridize to its complementary probe. To identify suitable indels, the genome sequences of the *japonica *rice cultivar Nipponbare and the *indica *cultivar 93-11 were aligned. The sequences used in the alignment are the pseudomolecules of cv. Nipponbare assembled by TIGR (version 3) [49], based on the International Rice Genome Sequencing Project (IRGSP) finished quality sequence , and the contigs from the whole-genome shotgun sequence of cv. 93-11 [19]. Whole-genome alignments were done using MUMmer and NUCmer 3.18 [21-23], with a 1000 base alignment extension and 1000 base maximum gap length. Indels shorter than 30 bases were excluded from further analysis, because they do not meet the probe length requirements. Using Perl scripts, the indel sequences were first masked for simple repeats. Next, the indel sequences were masked for complex repeats following Blast analysis [50] against a collection of known rice repetitive elements [24]. Indels with sufficiently long stretches of unique sequence (at least 30 nucleotides) were considered for probe design.

### Oligonucleotide probe design

Oligonucleotide probes were designed to be complementary to the indel sequences. Long oligonucleotides (68–70 bases) were used to ensure sufficient signal intensities [51] following hybridization. For uniformity in hybridization, probes were selected to have a balanced GC content with an optimum melting temperature (Tm) of 83°C and a range between 78°C and 88°C calculated using the "irreversible" formula for oligonucleotides greater than 50 bases [52]. To ensure that the probes do not cross-hybridize with any other sequences in the genome, potential probes were excluded that had greater than 70% identity across the entire probe, or stretches of contiguous sequence longer than 20 bases with 100% identity to another sequence in the genome [53]. The probes were designed to overlap a maximum of 20 bases of sequence extending beyond the indel on each side. Therefore, for a 70 mer, the minimum indel size is 30 bases. Perl scripts were employed for processing the indel sequences for oligonucleotide probe design based on the established parameters. Potential probe sequences were checked using Blast searches of the whole genome sequences of Nipponbare and 93-11 to exclude those with hits having a percent identity greater than 70%. The remaining probes were sorted by Tm, and the probe closest to the optimum was selected for each indel. The SFP discovery scripts can be found in Additional File 5, and the output of those scripts in Additional File 6. A total of 880 putative SFPs were identified from indels with sequences meeting the probe design criteria (Additional File 1). Additionally, six probes were designed from sequences not present in rice to provide negative controls, and six probes were designed from sequences present in both Nipponbare and 93-11 for use as positive controls. Six probes were designed from repetitive sequences for optimization and troubleshooting of the hybridization protocol. Details of the various control elements are provided in Additional File 3.

### Microarray printing

The oligonucleotides were commercially synthesized (Operon Biotechnologies, Huntsville, AL) with a 5'-amine modification. The synthesized oligonucleotides were arranged in 384-well plates, and dissolved at 20 pmol/μL in 3× SSC buffer. The oligonucleotide probes were printed on Superamine substrate slides (SMM, Telechem, Sunnyvale, CA) using an Omnigrid 100 printer (Genomic Solutions,) Ann Arbor, MI equipped with Telechem SMP3 spotting pins. Each probe, including 880 putative markers and 18 controls, was printed with three replicates per slide in separate subarrays. After printing, the slides were baked at 80°C for two hours.

### Target preparation

DNA samples were extracted from leaf tissues using a modified chloroform-SDS protocol [54]. The genomic DNA at a concentration of 100 ng/ul in a volume of 100 ul was sheared using a High Intensity Ultrasonic Processor device (Sonics & Materials Inc., 250-Watt Model, Newtown, CT). For each sample, 1 μg of sheared DNA was labeled with Cy3 or Cy5 dUTP/dCTP (Amersham Biosciences, Piscataway, NJ) using the BioPrime Array CGH Genomic Labeling System (cat# 18095-012, Invitrogen, Carlsbad, CA) in a 50 μL volume with a 12–16 hour reaction time. The Cy3 and Cy5 labeled products were purified simultaneously in a single spin-column (Qiagen PCR purification kit, cat# 28104, Valencia, CA), and eluted in 25 μL of water.

### Hybridization

Prior to hybridization, the microarray slides were rehydrated over a 50°C water bath for ten seconds and then snap-dried on a 65°C heating block, repeating both steps four times. Next, the slides were UV cross-linked using a Stratalinker [180 mJ] (Stratagene, La Jolla, CA). The slides were then incubated in a 1% BSA solution in 6.6× SSC at 37°C for 40 minutes, placed in 1% SDS for five minutes, dipped ten times in water, and spun to dryness in a bench-top centrifuge at 1,000 rpm for 2 minutes. The hybridization buffer was prepared using 24 μL of labeled DNA (including both the Cy3 and Cy5 samples), 1.2 μL of 2% SDS, 3 μL of 20× SSC, and 1.8 μL of Liquid Block (Amersham Life Science, cat# 1059304). To denature the labeled samples, the hybridization buffer was heated in a thermocycler for five minutes at 100°C and placed immediately on ice. The hybridization buffer (60 ul) was loaded onto each slide under a cover slip (Lifter slip, Erie Scientific, 241x301-2-511) and incubated for 12–16 hours at 65°C in a hybridization chamber (Telechem/ArrayIt Hybridization Cassette, AHC). After hybridization, the slides were washed successively in three solutions for five minutes each, with 2× SSC and 0.5 % SDS at 65°C, 0.5× SSC at room temperature, and 0.2× SSC at room temperature. The slides were centrifuged to dryness at 1,000 rpm for 2 minutes.

### Data acquisition and analysis

The microarray slides were scanned with a Gene Pix Autoloader (Axon/Molecular Devices, 4200A01, Sunnyvale, CA) at a resolution of 10 μm per pixel with laser illumination (100% power) at 532 and 635 nm, and PMT gain settings between 700 and 800 (adjusted for balance between colors). The images were saved in 16-bit grayscale multi-image TIFF format. Spot finding and data extraction was done using GenePix Pro 6 software (Axon/Molecular Devices, Sunnyvale, CA) and a GAL format file describing the position and content of each spot created using the Gridder software connected to the Omnigrid 100 printer.

The extracted data were analyzed in the R statistical language  using the Limma package [55] of the BioConductor project [56]. Normalization for dye balance within arrays was done based on the color ratios of the non-polymorphic control spots and global loess normalization [57]. Replicate spots within arrays were handled using a correlation method [58]. A linear model was fitted to the log transformed color ratios of each probe, and an empirical Bayes approach was used to shrink the estimated sample variances towards a pooled estimate [59]. The R scripts used for the analysis are in Additional File 7.

### Experimental verification of SFPs

The SFPs were experimentally verified by hybridization with DNA from each of the sequenced cultivars on four slides. DNA samples from cv. Nipponbare and cv. 93-11 were each labeled with Cy5 and Cy3. A dye swap design was used with Nipponbare labeled with Cy3 and 93-11 labeled with Cy5 on two of the slides and 93-11 labeled with Cy5 and Nipponbare labeled with Cy5 on the other two slides. The slides were normalized for color balance using median centering.

### Polymorphism survey

A diverse panel of rice cultivars was genotyped using the microarrays. The panel included 21 accessions. There were four accessions from each of the five sub-populations of rice as previously established with microsatellite markers [27]. An accession of the Australian wild relative *Oryza meridionalis *was included as an out-group. Nipponbare DNA was used as a common reference, with one hybridization per genotype. The slides were normalized for color balance using median centering. SFP markers were scored as the 93-11 allele (different from the reference) for a log-fold change greater than 1, and scored as the Nipponbare allele for a log-fold change less than 0.5 (same as the reference). SFPs with intermediate log-fold changes were treated as missing data. Neighbor-joining trees were constructed using the neighbor-joining algorithm in Powermarker [60] based on a shared allele distance matrix, and visualized using TreeView [61]. Population structure was inferred and site-by-site probabilities for the population of origin of alleles were calculated with the model-based clustering method STRUCTURE [26], using the linkage ancestry model with a burn-in of 100,000 and 100,000 MCMC replications. Site-by-site probabilities of alleles were plotted using GGT [62].

### Bulked segregant analysis

A RIL population of 215 individuals derived from the blast resistant *indica *variety Sanhuangzhan 2 (SHZ) and the blast susceptible *japonica *variety Lijiangxin-tuan-heigu (LTH) was previously phenotyped for blast resistance [33]. The SHZ and LTH parents were genotyped by hybridization to the microarrays with a dye-swap on two slides to determine which SFP markers are polymorphic in the RIL population. DNA samples from individual RILs were divided into two pools with 73 individuals each according to their levels of blast resistance. A dye swap design was used with six slides. The data were lowess normalized using all features. The chromosomal positions of the markers were assigned according to their locations on the rice pseudomolecules.

## Abbreviations

Indel: insertion/deletion

## Competing interests

The authors declare that they have no competing interests.

## Authors' contributions

JDE carried out the bioinformatics for indel discovery, design of the oligonucleotide probes, and analysis of the microarray data, and drafted the manuscript, JJ developed the genomic labeling procedure, carried out the hybridization experiments, and drafted the materials and methods portion of the manuscript, MS designed primers surrounding the SFPs, and optimized a low-cost method for labeling of genomic DNA, ABK helped develop the procedure for labeling of genomic DNA and helped draft the manuscript, BL and HW produced the mapping populations, carried out the phenotyping experiment, and helped draft the manuscript, DWG conceived of the study, and participated in its design and coordination and helped to draft the manuscript.

**Figure 6:**
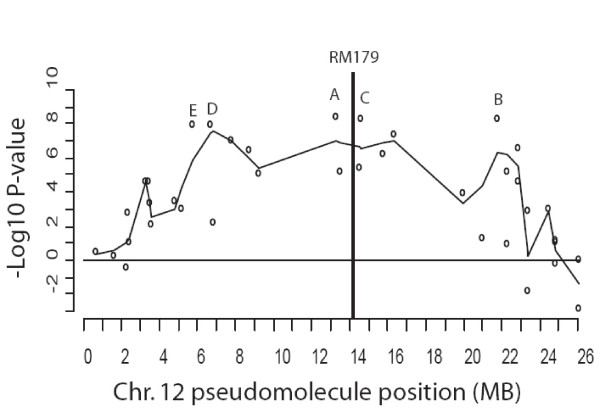
**Location of the five most significant SFP markers (ordered A-E) associated with blast resistance in the bulked segregant experiment**. The SFP markers are co-located with the previously mapped microsatellite marker (RM179), known to be linked to blast resistance.

## Supplementary Material

Additional File 1 Genomic locations of the 880 SFP oligonucleotide probes. Click here for file

Additional File 2 **SFP genotype scores for rice accessions**. A score of "1" indicates presence of the allele that is complementary to the probe sequence and a score of "0" indicates absence. Missing data are indicated by a "?" character. Click here for file

Additional File 4 Low Cost Labeling Method. Click here for file

Additional File 3 Sources of the positive, negative, and multi-copy control oligonucleotide probes. Click here for file

Additional File 5 SFP discovery scripts. Click here for file

Additional File 6 Output of SFP discovery scripts. Click here for file

Additional File 7 R-scripts for data analysis. Click here for file
