# Supplementary material for: Development and evaluation of a high-throughput, low-cost genotyping platform based on oligonucleotide microarrays in rice
Source: Plant Methods. 2008 May 29;4:13. doi: 10.1186/1746-4811-4-13 (PMC2435114; doi:10.1186/1746-4811-4-13)
Supplement: Additional File 4 — Low Cost Labeling Method. [file 1746-4811-4-13-S4.doc]

**A low-cost method of labeling total genomic DNA for microarray-based genotyping on multiplexed slides.**

Galbraith Laboratory, University of Arizona

Written by: Jeremy Edwards

and Megan Sweeney

[edwardsjd@ufl.edu](mailto:edwardsjd@ufl.edu)

[msweeney@cals.arizona.edu](mailto:msweeney@cals.arizona.edu)

**Restriction/Ligation**

**Materials**

*Mse*I Forward Adaptor 5’-GACGATGAGTCCTGAG-3’

*Mse*I Reverse Adaptor 5’-TACTCAGGACTCAT-3’

*Mse*I restriction enzyme 10 U/µL Fermentas - **#ER0982**

T4 ligase 5 U/µL Fermentas - **#EL0014**

T4 ligase buffer 10X Supplied with **#EL0014**

NaCl 0.5M

BSA 1mg/µL Fermentas - #B14

**Adaptor preparation**

250 µL Forward *Mse*I Adaptor @ 100 µM

250 µL Reverse *Mse*I Adaptor @ 100 µM

500 µL Total @ a final concentration of 50 µM each

Vortex. Then heat at 95˚C for 5 min to denature, and allow to cool slowly in a Styrofoam box to renature completely.

**Enzyme Master Mix**

T4 ligase buffer 10X 0.1 µL

NaCl 0.5M 0.1 µL

BSA @ 1mg/ml 0.05 µL

MseI (Tru): 1 unit (10 U/uL) 0.1 µL

T4 ligase: 1 unit (5 U/uL) 0.2 µL

Distilled water 0.45 µL

Total per tube: 1 µL

**Restriction/Ligation Reaction**

T4 buffer 10X 1 µL

NaCl 0.5M 1 µL

BSA @ 1mg/ml 0.5 µL

Enzyme master mix 1 µL

Mse adapter 2 µL

Template DNA (10ng/µl) 5.5 µL

Total per tube: 11 µL

Incubate at 37˚C for 2 hours

Dilute 10 fold by adding 90 µL of TE_01_ (10 mM tris.Cl, pH 7.5, containing 0.1 mM EDTA).

**Whole genome amplification/labeling**

**Preamplification**

Taq DNA pol 5U/µL Fermentas # **EP0406**

10X Taq Buffer with (NH_4_)_2_SO_4_ - MgCl_2_ Supplied with # **EP0406**

MgCl_2_ (25mM) Supplied with # **EP0406**

dNTPs, 2mM each Fermentas **#R0241 or #R0242**

MseI universal - unlabeled 20µM 5’-GATGAGTCCTGAGTA-3’

Reagent Final conc. Volume

Taq Buffer 10X 1X 2 µL

Taq DNA pol 5U/ul 5 U 0.2 µL

MgCl_2_ (25mM) 1.5mM 1.2 µL

dNTPs 2mM each 0.2mM 2 µL

Primer-MseI-unlabeled 20uM 1.6µM 3.2 µL

Diluted restriction/ligation product 2.4 µL

dd H_2_O 9 µL

Total 20 µL

PCR profile

1. 72˚C 5min*

2. 94˚C 30 sec

3. 54˚C 30 sec

4. 72˚C 2 min

5. Go to Step 2 for 29 cycles

6. 60˚C 10 min

7. 4˚C hold

*The initial 72˚C step is required for Taq polymerase to ligate the second strand. T4 DNA ligase only ligates one of the strands to the adaptor. Do not use a hot-start or hot-start DNA polymerases such as AmpliTaq Gold.

Dilute by adding 180 µL of TE_01_

Use the MseI universal primer 5’-GATGAGTCCTGAGTA-3’ labeled with Alexa Fluor 555 for sample DNA and Alexa Fluor 647 for control DNA.

**Amplification with labeling**

Reagent Final conc. Volume

Taq Buffer 10X 1X 5 µL

Taq DNA pol 5U/µL 2.5 U 0.5 µL

MgCl_2_ (25mM) 1.5 mM 3 µL

dNTPs 2mM each 0.2 mM 5 µL

Primer-*Mse*I-labeled @ 20µM 1.6 µM 4 µL

Diluted preamplification product 6 µL

dd H2O 26.5 µL

Total 50 µL

Important: Protect labeled primers and labeled PCR products from light.

PCR profile

1. 95˚C 3min

2. 94˚C 30 sec

3. 54˚C 30 sec

4. 72˚C 2 min

5. Go to Step 2 for 29 cycles

6. 60˚C 10 min

7. 4˚C hold

Purify samples using a MinElute 96 UF PCR Purification Kit (Qiagen cat# 28051 for 4 or 28053 for 24). Elute in 20 µL of water. Use the Nanodrop to check for consistency of labeling and dye balance. Store at 4˚C protected from light.

**Hybridization**

Slide Preparation (can be done at any time prior to hybridization)

1. Re-hydrate slide over a hot water bath for 10 sec.
   1. Hold slide with the label side down over the water vapor.
   2. Watch spots carefully so that they do not over-hydrate (this will cause them to begin to merge together).
2. Snap dry the slide on a 65˚C heating block for 5 sec.
   1. Place slide label side up on heating block.
   2. Allow slide to cool for 1 min.
3. Repeat steps 1-3 for a total of four times.

(The rehydration step is important to obtain uniform spots without a doughnut effect; however if you feel uncomfortable with performing the rehydration step, you can proceed directly to UV cross linking)

1. UV cross-link the slides by exposing them, label side up, to 60mJ in a Stratalinker cross-linker.
2. Wash the slide in 1% SDS (prepared in sterile DDH_2_O) for 5 min at RT on a shaker or agitate by hand.
3. Remove SDS by dipping the slides ten times into sterile DDH_2_O.
4. Immediately transfer the slides to 100% ethanol, dip five times, then incubate for three min with shaking.
5. Spin dry slide in centrifuge at no more than 200 x g for 2-4 min.
6. Repeat ethanol wash if any visible streaks remain after spin dry.
7. Slides can be stored in a lint-free light-proof box at RT with low humidity.
8. Place slides in the hybridization cassette. Preheat the cassette to 55C

**Hybridization**

Oligo aCGH/ChIP-on-Chip Hybridization Kit

Agilent #5188-5220 or #5188-5380 (large volume)

**Hybridization master mix**

Labeled samples + ddH_2_O 66.5 µL

20X SSC 7.5 µL

2% SDS 5 µL

2X Hi-RPM Hybridization Buffer 12.5 µL

10X Oligo aCGH Blocking Agent 2.5 µL

Liquid Block 6 µL

Total 100 µL

Denature the labeled samples in the hybridization buffer by heating in a thermocycler for five minutes at 95˚C and place immediately on ice.

Add the samples to the slide. Add H_2_O to any blank wells. Place grey rubber stopper on top of the wells. Wrap the cassette in a damp paper towel and cover with foil. Place cassette on a shaker within the incubator. Incubate at 65˚C for 24 hours.

Prepare the following solutions:

2x SSC, 0.5% SDS @ 55˚C

0.5x SSC @ RT

0.05x SSC @ RT

Quickly disassemble the multiplex cassette and place the slide in the first wash buffer.

Wash slide in the each solution in order for 5 min

Washing is done by immersing the slides in a glass slide-staining jar containing the appropriate volume of wash buffer, followed by placing it on a belly shaker at 60 rpm. Pre-heat the first wash solution, and make sure the slides are completely immersed in wash buffer.

After completion of the washes, spin dry the slide in centrifuge at no more than 1,000 rpm for 2-4 min.

**References**

Wolf lab AFLP protocol: <http://bioweb.usu.edu/wolf/aflp_protocol.htm>

Vos, P., R. Hogers, M. Bleeker, M. Reijans, T. van de Lee, M. Hornes, A. Frijters, J. Pot, J. Peleman, M. Kuiper, and et al. 1995. AFLP: a new technique for DNA fingerprinting. *Nucleic Acids Res* **23:** 4407-4414.

Casa, A.M., C. Brouwer, A. Nagel, L. Wang, Q. Zhang, S. Kresovich, and S.R. Wessler. 2000. Inaugural article: the MITE family heartbreaker (Hbr): molecular markers in maize. *Proc Natl Acad Sci U S A* **97:** 10083-10089.

**Cost Analysis for Microarray Genotyping**

Total for One Sample: $17.74

**Restriction/Ligation Reaction**

*Mse*I restriction enzyme 10 u/ul (Fermentas - **#ER0982**) $144.00/1,500U

Need 1U = 1500 reactions/tube

Item = $0.10

T4 ligase 5 u/ul (Fermentas - #**EL0014**) $40.00 200U

Need 1 U = 200 reactions/ tube

Item = $0.20

**First round PCR amplification:**

Taq DNA polymerase (Fermentas - #**EP0406**) $670.00 for 5,000U

Need 1U = 5,000 reactions/tube = $0.13 per sample

Control DNA gives enough for 33 second round reactions add $0.01

Item = $0.14

**Second round PCR:**

Taq DNA Polymerase

$670.00 for 5,000U

Need 2.5U = 2,000 reactions/tube

= $0.335 for each reaction, four reactions/ sample

Item = $1.34

Labeled Primers

$774.00 for 1µmol scale with Alexa Fluor modification, 50µmol after purification

Need 4 µL of 20µM = 0.08nmol

= 625 reactions/ tube; need four labeling reaction/sample

Item = $4.95

Column Purification

MinElute 96 UF PCR Purification Kit (24) Qiagen cat #28053   $874

24 x 96 = 2304 = 0.38/well need two per hyb, four per sample

Item = $1.52

dNTPs

Invitrogen Cat # 18427-088, $349.00 for 1mL of 10mM.

Need 5ul of 2mM = 1,000 reactions/tube need four reactions/sample

Item = $1.40

**Hybridization**

HiSens Slide

Schott $15.30/slide, 24 subarrays/ slide

Need 2 subarrays/ sample

Item = $1.28

Printing of subarrays

$3/subarray, need 2 subarrays/sample

Item = $6

Agilent Genomic Hybridization Buffer

Agilent Genomic Hyb Buffer #5188-5380 $814.30 for 25 mL

Need 12.5 µL/ hybridization, = 2,000 hybs/ bottle

2 hybs/sample

Item = $0.81
