# Supplementary material for: Development and evaluation of a high-throughput, low-cost genotyping platform based on oligonucleotide microarrays in rice
Source: Plant Methods. 2008 May 29;4:13. doi: 10.1186/1746-4811-4-13 (PMC2435114; doi:10.1186/1746-4811-4-13)
Supplement: Additional File 3 — Sources of the positive, negative, and multi-copy control oligonucleotide probes. [file 1746-4811-4-13-S3.doc]

Supplementary Table 2. Sources of the positive, negative, and multi-copy control oligonucleotide probes.

| **Probe** | **Status** |
| --- | --- |
| BA1 | absent in *O. sativa* |
| ETR3 | absent in *O. sativa* |
| FLC | absent in *O. sativa* |
| FRI | absent in *O. sativa* |
| ORFX | absent in *O. sativa* |
| TB1 | absent in *O. sativa* |
| Adh1 | single copy in *O. sativa* |
| MOC1 | single copy in *O. sativa* |
| OsTB1 | single copy in *O. sativa* |
| Pi-ta | single copy in *O. sativa* |
| Se1 | single copy in *O. sativa* |
| Waxy | single copy in *O. sativa* |
| mPing | multiple copies in *O. sativa* |
| Osr13_Tos5 | multiple copies in *O. sativa* |
| Osr25_Dashdeng | multiple copies in *O. sativa* |
| Osr27_Rire9 | multiple copies in *O. sativa* |
| Osr32 | multiple copies in *O. sativa* |
| Osr44 | multiple copies in *O. sativa* |
